# Preliminary analysis of gut microbiome characteristics in children with obstructive sleep apnea hypopnea syndrome

**DOI:** 10.3389/fneur.2025.1615891

**Published:** 2025-11-14

**Authors:** Zhihui Wang, Luting Zhou, Yanyu He, Xueyun Xv, Meng Lv, Zhen Zhang, Fengqian Wang, Shuqi Wang, Yuqing Wang

**Affiliations:** 1 Department of Respiration, Children’s Hospital of Soochow University, Suzhou, China; 2 Jinan Maternity and Child Care Hospital Affiliated to Shandong First Medical University, Jinan, China

**Keywords:** children, obstructive sleep apnea hypopnea syndrome, gut microbiome, polysomnography, high-throughput nucleotide sequencing

## Abstract

**Objective:**

To analyze the characteristic changes in the gut microbiome in children with obstructive sleep apnea-hypopnea syndrome (OSAHS) and to investigate the relationship between the gut microbiome and polysomnography (PSG) results.

**Methods:**

Children diagnosed with primary snoring and OSAHS by PSG were enrolled in the study group. Nonsnoring children undergoing elective surgery were selected as the control group. Stool, sleep monitoring data, and medical history data were collected. The clinical history data were analyzed by SPSS 25.0 software. 16S rRNA high-throughput sequencing technology was used to analyze the gut microbiome, and relevant biostatistical methods were used to analyze and describe the characteristics of the gut microbiome.

**Results:**

A total of 62 OSAHS patients (42 mild OSAHS and 20 moderate to severe OSAHS), 16 primary snoring patients and 46 controls were enrolled in this study. There were significant differences in the partial alpha diversity index (observed otus index, Chao1 index) and beta diversity under the Jaccard and unweighted UniFrac distance methods between the mild OSAHS group and the moderate to severe OSAHS group. There were differences in some gut microbiome at different levels of phylum, class, order, family, genus and species between the control group and OSAHS group. There was a significant difference in the abundance ratio between Firmicutes and Bacteroidetes (F/B), and the ratio gradually increased among the three groups. The predictive model for OSAHS diagnosis established by the receiver operating characteristic (ROC) curve showed that the area under the curve (AUC) of Firmicutes and the F/B were more than 50%. At the genus level, Akkermansia was positively correlated with sleep efficiency (SE), Dialister was positively correlated with mean oxygen saturation (SaO_2mean_) and lowest oxygen saturation (LSaO_2_), Escherichia-Shigella was negatively correlated with total sleep time (TST), and Faecalibacterium was negatively correlated with the obstructive apnea index (OAI).

**Conclusion:**

The gut microbiome of children with OSAHS is slightly different at the phylum, class, order, family, genus and species levels. The F/B and Firmicutes abundance detection have limited predictive capability for the diagnosis of OSAHS. At the genus level, some gut microbiota were correlated with PSG indicators.

## 1 Introduction

Obstructive sleep apnea-hypopnea syndrome is a disease in which the upper airway repeatedly collapses during sleep, affecting sleep structure and normal ventilation and leading to pathophysiological changes.

OSAHS in children is a multifactorial disorder influenced by anatomical, neuromuscular, and systemic factors. Among these, obesity has been shown to play a pivotal role not only by mechanically narrowing the upper airway but also through its effects on ventilatory control and systemic inflammation. These mechanisms have been extensively discussed in the context of obesity hypoventilation syndrome, which shares overlapping features with pediatric OSAHS in terms of pathophysiology and ventilatory impairment (1). Given the emerging role of the gut–lung axis, it is plausible that obesity-driven alterations in immune and metabolic homeostasis may also influence the gut microbiota, thereby contributing to the progression or phenotype of OSAHS in children.

It can cause a series of complications, such as neurocognitive disorders, maxillofacial dysplasia, hypertension, cardiovascular disease, and type 2 diabetes (2–4). Therefore, timely diagnosis and treatment are of great significance for improving the prognosis.

The normal and stable gut microbiome plays an important role in promoting digestion and absorption and substance metabolism and regulating the body’s immunity. Some studies have found that the gut microbiome is related to endocrine, cardiovascular, neurological, respiratory, and digestive system diseases (5, 6). Recently, the relationship between OSAHS and gut microbiome has become a hot topic. Some changes in gut microbiota have been found in animal experiments simulating sleep fragmentation and chronic intermittent hypoxia (7, 8). One study on the gut microbiota of adults with OSAHS showed an intestinal microecological imbalance in patients with OSAHS, mainly due to the reduction in the relative abundance of probiotics producing short-chain fatty acids and the increase in pathogenic bacteria (9). Collado et al. (10) found that the diversity and abundance of gut microbiome in children who snore were significantly lower than those in nonsnorers, but the subjects of this study did not undergo PSG.

Based on the above studies, we speculate that OSAHS can probably lead to changes in the abundance and composition of gut microbiota. Therefore, on the basis of clinical diagnosis by PSG monitoring, we analyzed the abundance, diversity and structure of gut microbiota in children with OSAHS and explored the relationship between gut microbiome and PSG monitoring indicators.

## 2 Methods

### 2.1 Subjects

This study was an observational case–control study conducted from November 2020 to September 2022 in the Children’s Hospital of Soochow University. Children aged 2 to 16 years with a chief complaint of snoring and/or mouth breathing during night sleep were enrolled, and PSG monitoring was completed at night.

Children, in the same age group, who underwent elective surgery during the same period and had no clinical manifestations of OSAHS were enrolled as the control group.

Patients with the following conditions were excluded: children with inflammatory bowel disease or recent gastrointestinal dysfunction. Use of antibiotics in the past 60 days, probiotics in the past 30 days, or anti-inflammatory drugs in the past 15 days.

The study was approved by the ethics committee of Children’s Hospital of Soochow University (No: 2022CS090).

### 2.2 PSG monitoring

All patients underwent full-night polysomnography under the supervision of professionals in a sleep laboratory (Compumedics Grael). The PSG data collected included obstructive apnea hypopnea index (OAHI), TST, SE, no rapid eye movement (NREM), rapid eye movement (REM), oxygen desaturation index (ODI), LSaO_2_, SaO_2mean_, OAHI, OAI, and apnea hypopnea index (AHI).

Diagnostic criteria of OSAHS in children (3) OAHI was defined as the sum of obstructive apnea events, mixed apnea events, and obstructive hypoventilation occurring, on average, every hour of sleep per night, and OAHI >1 event/h is recommended as the standard diagnosis of OSAHS in children. Mild OSAHS was defined as 1 event/h < OAHI ≤5 events/h; moderate OSAHS was defined as 5 events/h < OAHI ≤10 events/h; and severe OSAHS was defined as OAHI >10 events/h.

### 2.3 Stool sample collection

Stool collection tubes were distributed to the parents, and stool samples were collected cleanly, no less than 100 mg. Within 4 h, it was transferred to an ultralow temperature refrigerator at −80 °C and was frozen. Stool samples were collected at admission from children undergoing elective surgery.

### 2.4 Microbiome assessment

All fecal samples were tested for 16S rDNA by Lianchuan Biotechnology Co., Ltd. (Hangzhou, China). The main testing steps were as follows: DNA from different samples was extracted using CTAB according to the manufacturer’s instructions. DNA samples were amplified by polymerase chain reaction (PCR) using bar-coded primers flanking the V3–V4 [341F (5′-CCTACGGGNGGCWGCAG-3′), 805R (5′-GACTACHVGGGTATCTAATCC-3′)] region of the 16S rRNA gene. The PCR products were confirmed with 2% agarose gel electrophoresis.

The PCR products were purified by AMPure XT beads (Beckman Coulter Genomics, Danvers, MA, USA) and quantified by Qubit (Invitrogen, USA). The amplicon pools were prepared for sequencing, and the size and quantity of the amplicon library were assessed on an Agilent 2100 Bioanalyzer (Agilent, USA) and with the Library Quantification Kit for Illumina (Kapa Biosciences, Woburn, MA, USA), respectively. The libraries were sequenced on the NovaSeq PE250 platform.

### 2.5 Data analysis

Samples were sequenced on an Illumina NovaSeq platform according to the manufacturer’s recommendations provided by LC-Bio. Paired-end reads were assigned to samples based on their unique barcode and truncated by cutting off the barcode and primer sequence. Paired-end reads were merged using FLASH. Quality filtering of the raw reads was performed under specific filtering conditions to obtain high-quality clean tags according to fqtrim (v0.94). Chimeric sequences were filtered using Vsearch software (v2.3.4). After dereplication using DADA2, we obtained a feature table and feature sequence.

Then, according to the SILVA (release 138) classifier, feature abundance was normalized using the relative abundance of each sample. Alpha diversity and beta diversity were calculated by QIIME2. Blast was used for sequence alignment, and the feature sequences were annotated with the SILVA database for each representative sequence. Taxonomic differences were analyzed using LEfSe (linear discriminant analysis effect size) with an LDA score >2 as the significance cutoff. The relationship between the overall gut microbiota and PSG indices was analyzed by RDA. The relationship between gut microbiome and PSG indices was analyzed by Spearman correlations. The receiver operating characteristic (ROC) curves of OSAHS diagnosis and prediction were drawn by SPSS 25.0 software.

### 2.6 Statistical analyses

SPSS 25.0 software was used to analyze the clinical data. The Mann–Whitney U test was used to compare the differences between the two groups. The Kruskal–Wallis test was used for comparisons between multiple groups. The chi-square test was used for gender. For all results, *p* < 0.05 was considered statistically significant.

## 3 Results

### 3.1 Demographic data of enrolled children

A total of 124 patients were included in the study, consisting of 62 patients with OSAHS (42 patients in the mild OSAHS group, 20 patients in the moderate to severe OSAHS group), 16 patients with primary snoring, and 46 controls. There was no significant difference in age, sex or BMI among the three groups (*p* > 0.05) (Tables 1, 2).

**Table 1:** Demographic data of OSAHS group, primary snoring group and control group.

	OSAHS group	Primary snoring group	Control group	*H*/*χ*^2^	*p*
Number	62	16	46	–	–
Age (years)	4 (6, 9)	5 (7, 8)	4 (5, 9)	1.455^a^	0.483
Sex (male/female)	48/14	10/6	32/14	1.757^b^	0.415
BMI (kg/m^2^)	16.5 (14.6, 20.8)	14.6 (15.4, 20.1)	15.0 (16.2, 17.9)	1.714^a^	0.425

BMI, body mass index.

a Kruskal-Wallis H test.

b Chi-square test.

**Table 2:** General data of mild OSAHS group moderate to severe OSAHS group moderate to severe OSAHS group.

	Mild OSAHS group	Moderate to severe OSAHS group	*Z*/*χ*^2^	*p*
Number	42	20	–	–
Age (years)	5.0 (6.5, 9.0)	3.3 (5.0, 8.3)	−1.719^a^	0.086
Sex (male/female)	32/10	16/14	0.00^b^	0.99
BMI (kg/m^2^)	15.2 (16.6, 20.8)	14.1 (15.8, 22.4)	−0.738^a^	0.461

BMI, body mass index.

a Mann–Whitney U test.

b Chi-square test.

There were significant differences in the (ODI, LSaO_2_, SaO_2mean_, OAHI, OAI, and AHI) among the primary snoring group, mild OSAHS group and moderate to severe OSAHS group (*p* < 0.05), while there were no significant differences in other indicators (TST, SE, NREM, and REM) (Table 3).

**Table 3:** Sleep monitoring results of children with mild OSAHS, moderate to severe OSAHS and simple snoring.

	Mild OSAHS	Moderate to severe OSAHS	Simple snoring	*H*	*p*
TST (min)	440.25 (396.75, 479.63)	445.75 (420.75, 495.13)	454.50 (424.13, 504.63)	2.07	0.36
SE (%)	84.55 (73.40, 93.10)	83.25 (80.33, 90.08)	87.40 (75.20, 90.90)	0.24	0.89
NREM (%)	80.60 (76.08, 86.23)	78.20 (74.65, 83.98)	81.75 (78.28, 84.03)	2.12	0.35
REM (%)	19.40 (13.78, 23.60)	21.80 (16.00, 25.38)	18.25 (16.00, 21.80)	2.43	0.23
ODI (>4%)	0.50 (0.10, 1.23)	5.20 (1.43, 9.25)	0.10 (0.00, 0.425)	33.75	<0.01
LSaO_2_ (%)	91.00 (88.00, 93.00)	83.00 (73.25, 88.00)	93.00 (91.00, 94.00)	33.59	<0.01
SaO_2mean_ (%)	97.00 (96.00, 98.00)	96.00 (95.25, 97.00)	98.00 (97.00, 98.00)	8.94	0.01
OAHI (events/h)	2.35 (1.55, 3.33)	12.15 (6.40, 16.03)	0.60 (0.13, 0.70)	63.08	<0.01
OAI (events/h)	0.00 (0.00, 0.10)	1.95 (0.03, 5.40)	0.00 (0.00, 0.00)	27.01	<0.01
AHI (events/h)	1.75 (1.30, 2.93)	5.85 (1.68, 15.88)	0.60 (0.13, 0.70)	38.07	<0.01

TST, total night sleep time; SE, sleep efficiency; NREM, no rapid eye movement; REM, rapid eye movement; ODI, oxygen desaturation index; LSaO_2_, lowest oxygen saturation; SaO_2mean_, the mean oxygen saturation; OAHI, obstructive apnea hypopnea index; OAI, obstructive apnea index; AHI, apnea hypopnea index; Kruskal–Wallis H test.

### 3.2 Biological analysis

#### 3.2.1 Analysis of diversity

Analysis of alpha diversity: The Good’s coverage index of the control group, primary snoring group and OSAHS group was significantly close to 1, and there was no significant difference among the other indices. The observed otus and chao 1 index in the mild OSAHS group were significantly higher than those in the moderate to severe OSAHS group (*p* < 0.05).

**Figure 1:**
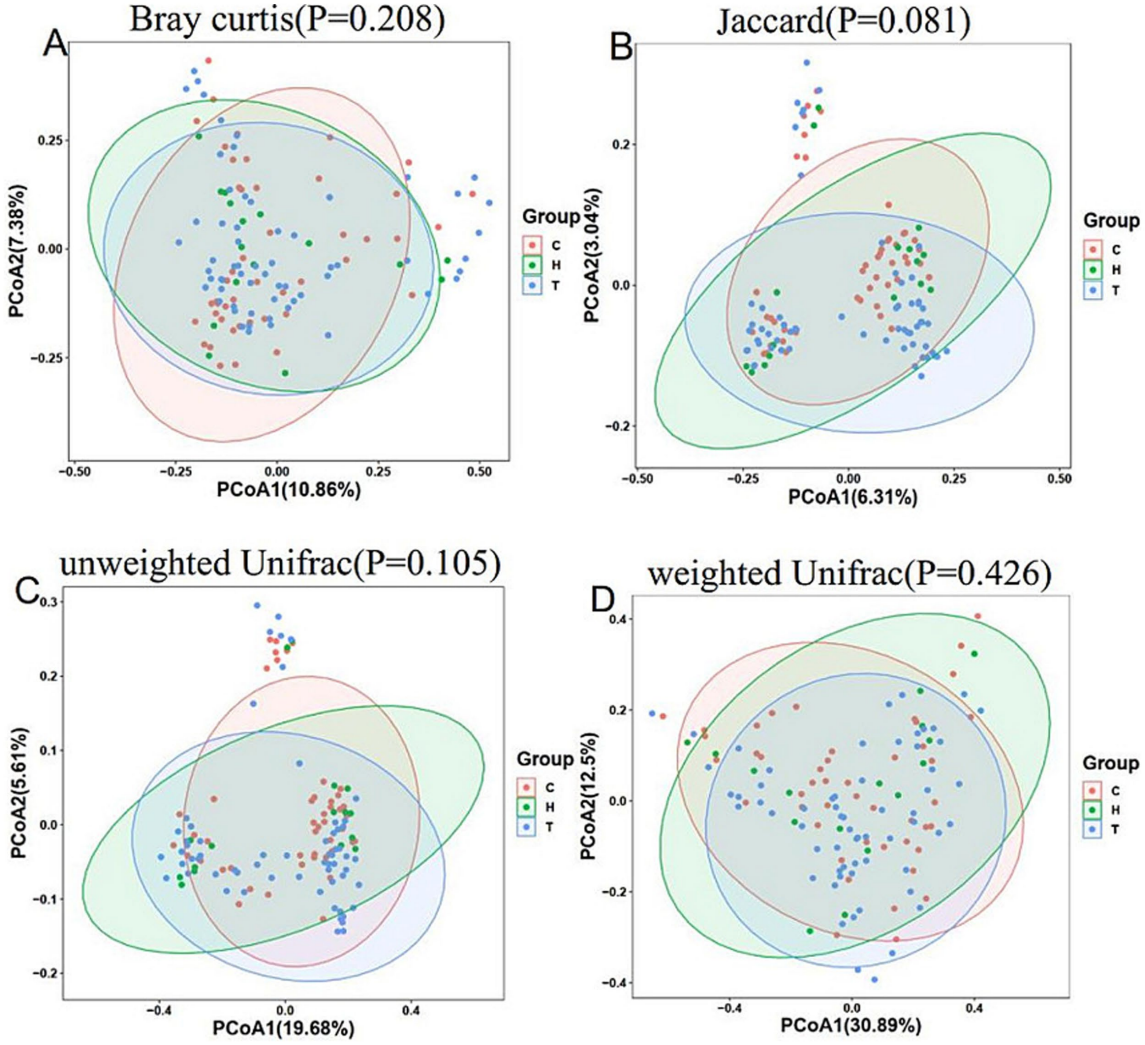
PCoA of control group, primary snoring group and OSAHS group. PCoA is analyzed through bray curtis **(A)**, jaccard **(B)**, unweighted unifrac **(C)**, and weighted unifrac **(D)** distance matrices. C, control group; T, OSAHS group; H, simple snoring group.

**Figure 2:**
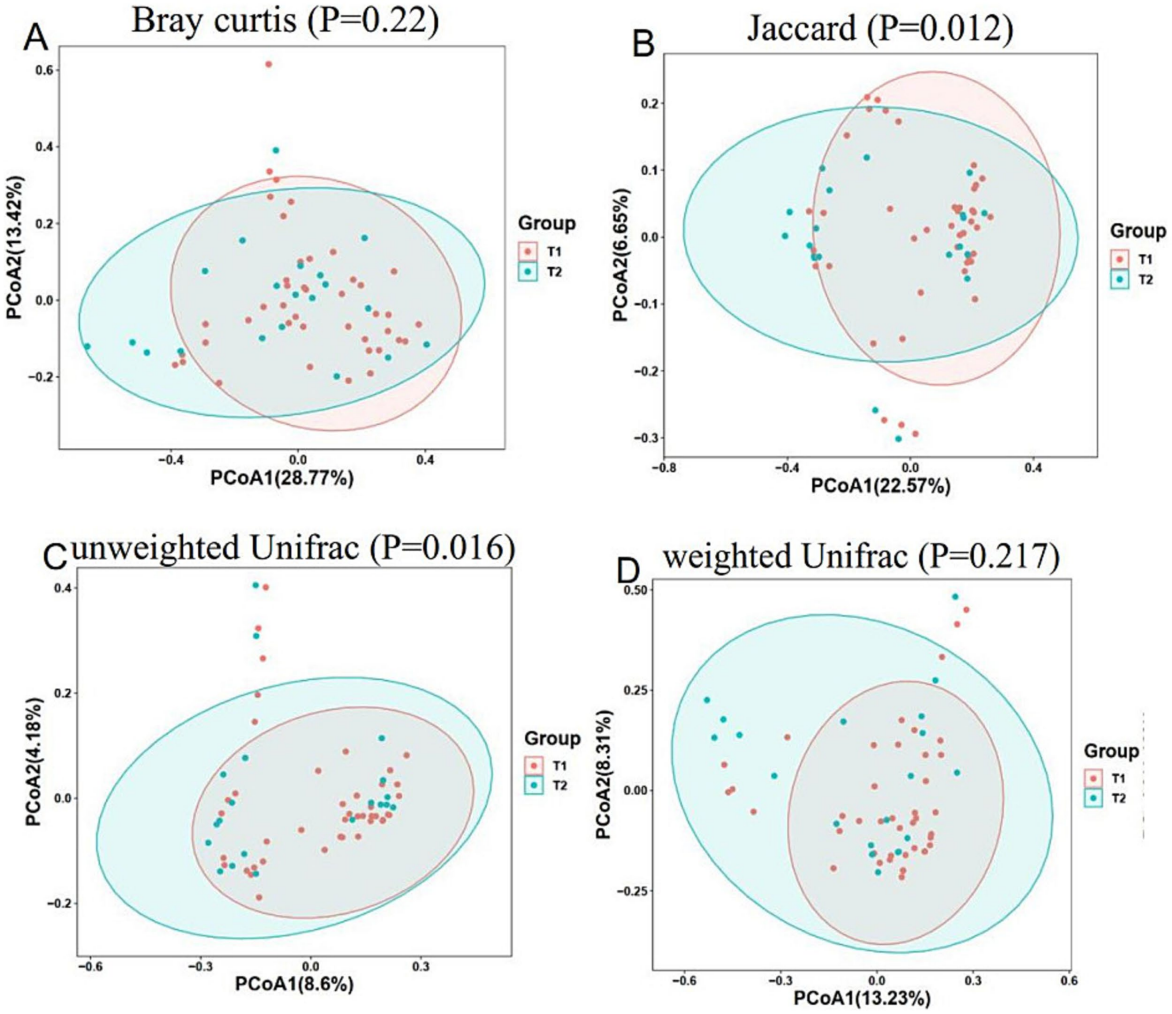
PCoA of mild OSAHS group and moderate to severe OSAHS group. PCoA is analyzed through bray curtis **(A)**, jaccard **(B)**, unweighted unifrac **(C)**, and weighted unifrac **(D)** distance matrices. T1, mild OSAHS group; T2, moderate to severe OSAHS group.

Analysis of beta diversity: In PCoA, there was no significant difference in distance among the control group, primary snoring group and OSAHS group (*p* > 0.05) (Figure 1). In the mild OSAHS group and the moderate to severe OSAHS group, the distance differences under the Jaccard and unweighted UniFrac distance methods were statistically significant (both *p* < 0.05). Therefore, the composition of the gut microbiome structure was different between the two groups (Figure 2).

#### 3.2.2 Species composition and diversity

**Figure 3:**
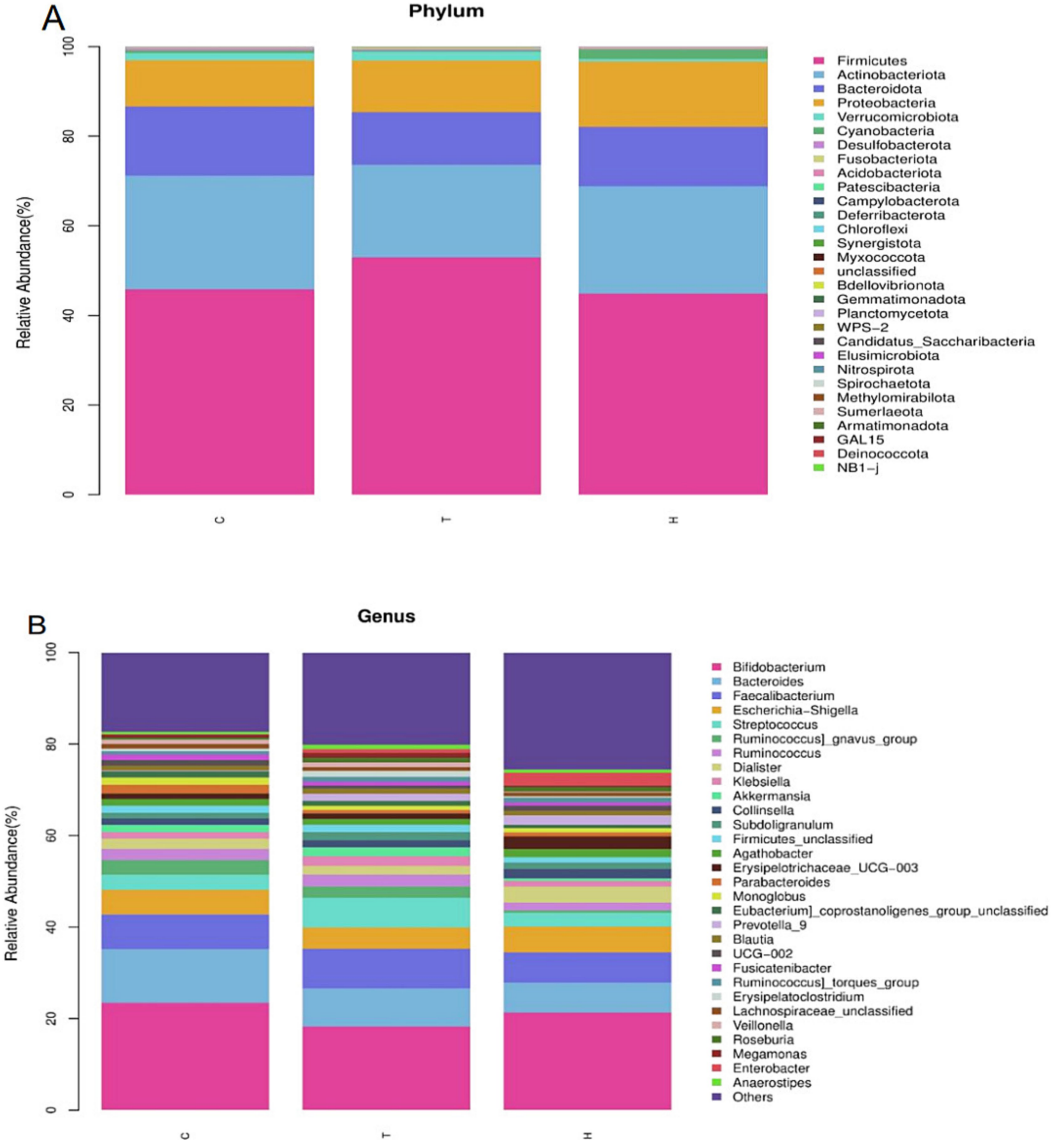
Histogram of species classification; Phylum **(A)** and Genus **(B)** levels. C, control group; T, OSAHS group; H, simple snoring group. In the figure, the horizontal axis is the grouping, and the vertical axis is the relative abundance of a bacterial group. Different colors correspond to different species at the same level.

At the phylum level, it was mainly composed of Firmicutes (45.84%), Actinobacteriota (25.34%), Bacteroidota (15.47%), Proteobacteria (10.33%) and Verrucomicrobia (1.57%). At the genus level, it was mainly composed of Bifidobacterium (23.44%), Bacteroides (11.72%), Faecalibacterium (7.65%), Escherichia-Shigella (5.31%) and Streptococcus (3.32%) (Figure 3).

**Figure 4:**
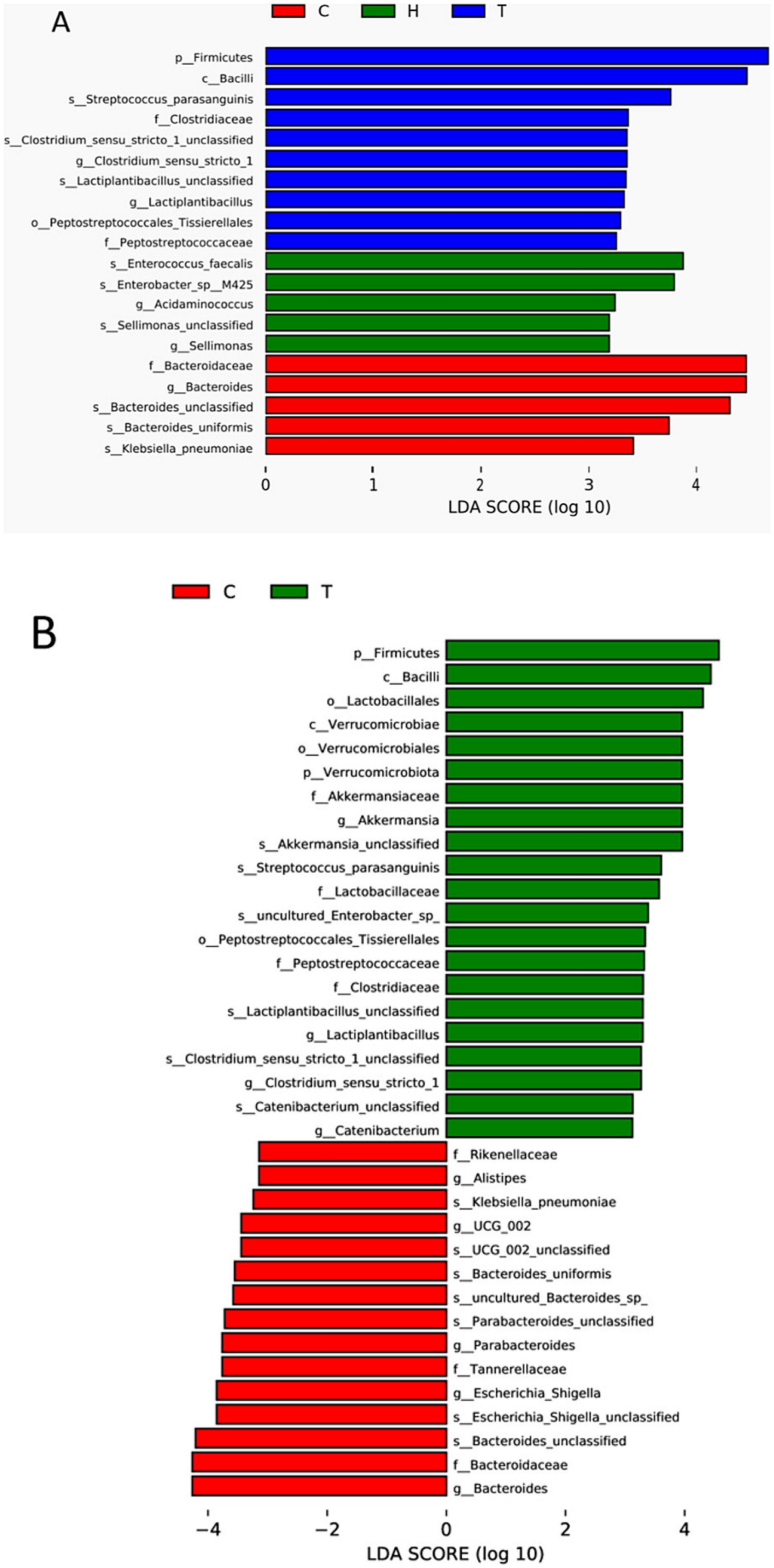
Histogram of the LDA distribution. The relative abundance of the most discriminative fecal metabolites was compared between groups according to LEfSe **(A,B)**. C, control group; T, OSAHS group; H, simple snoring group.

LEfSe analysis showed that at the phylum level, the abundance of Firmicutes in the OSAHS group was significantly higher than that in the primary snoring group and control group. At the genus level, the abundance of Bacteroides was significantly higher in the control group than in the other two groups (Figure 4).

Comparison of the top 10 abundant gut microbiome between the control group and the OSAHS group: At the phylum and genus levels, the abundance of Firmicutes, Verrucomicrobia, and Akkermansia in the OSAHS group was significantly higher than that in the control group, and the abundance of Bacteroides, Escherichia-Shigella, and Parabacteroides in the control group was significantly higher than that in the OSAHS group, all with statistical significance (LDA score >2) (Figure 4).

**Figure 5:**
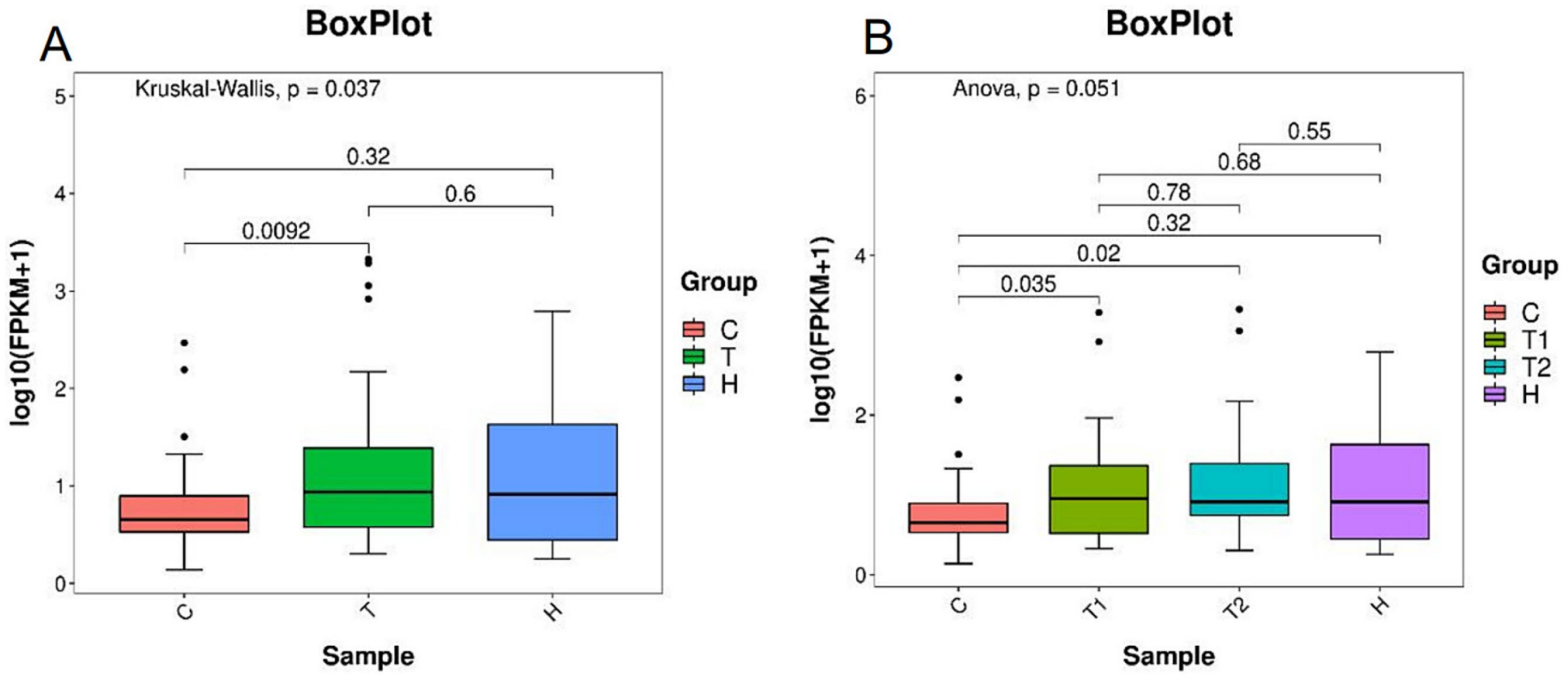
Ratios of Firmicutes and Bacteroidetes abundance **(A,B)**. C, control group; T, OSAHS group; H, simple snoring group; T1, mild OSAHS group; T2, moderate to severe OSAHS group.

At the phylum level, the F/B were significantly higher in the OSAHS group than in the control group (*p* < 0.05) (Figure 5).

#### 3.2.3 Analysis of gut microbiome and PSG indices

**Figure 6:**
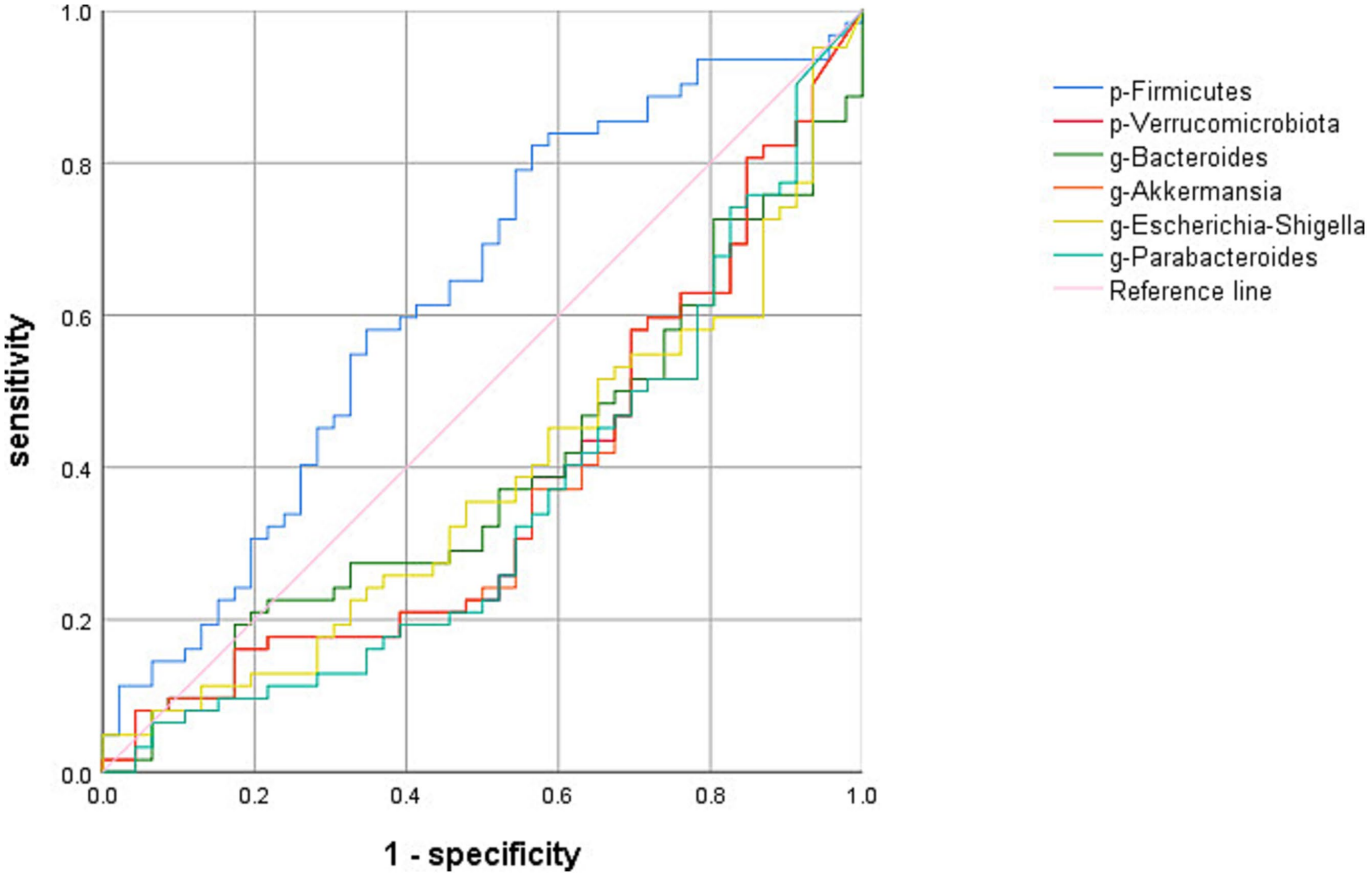
Receiver operating characteristic (ROC) curve analysis of the top 10 abundant gut microbiome with significant differences at phylum and genus level. “p” stands for phylum level; “g” stands for genus level; Firmicutes: AUC62.4%, Verrucomicrobia: AUC37.4%, Escherichia-Shigella: AUC37.9%, Parabacteroides: AUC35%, Akkermansia: AUC37.2%, Bacteroides: AUC38.8%.

The ROC of OSAHS diagnosis and prediction was established by the top 10gut microbiome with significant differences and abundance at the phylum and genus levels. Only Firmicutes had an ROC-AUC of 62.4%, a sensitivity of 82.3%, a specificity of 43.5%, and a cutoff value of 41.5 (Figure 6).

**Figure 7:**
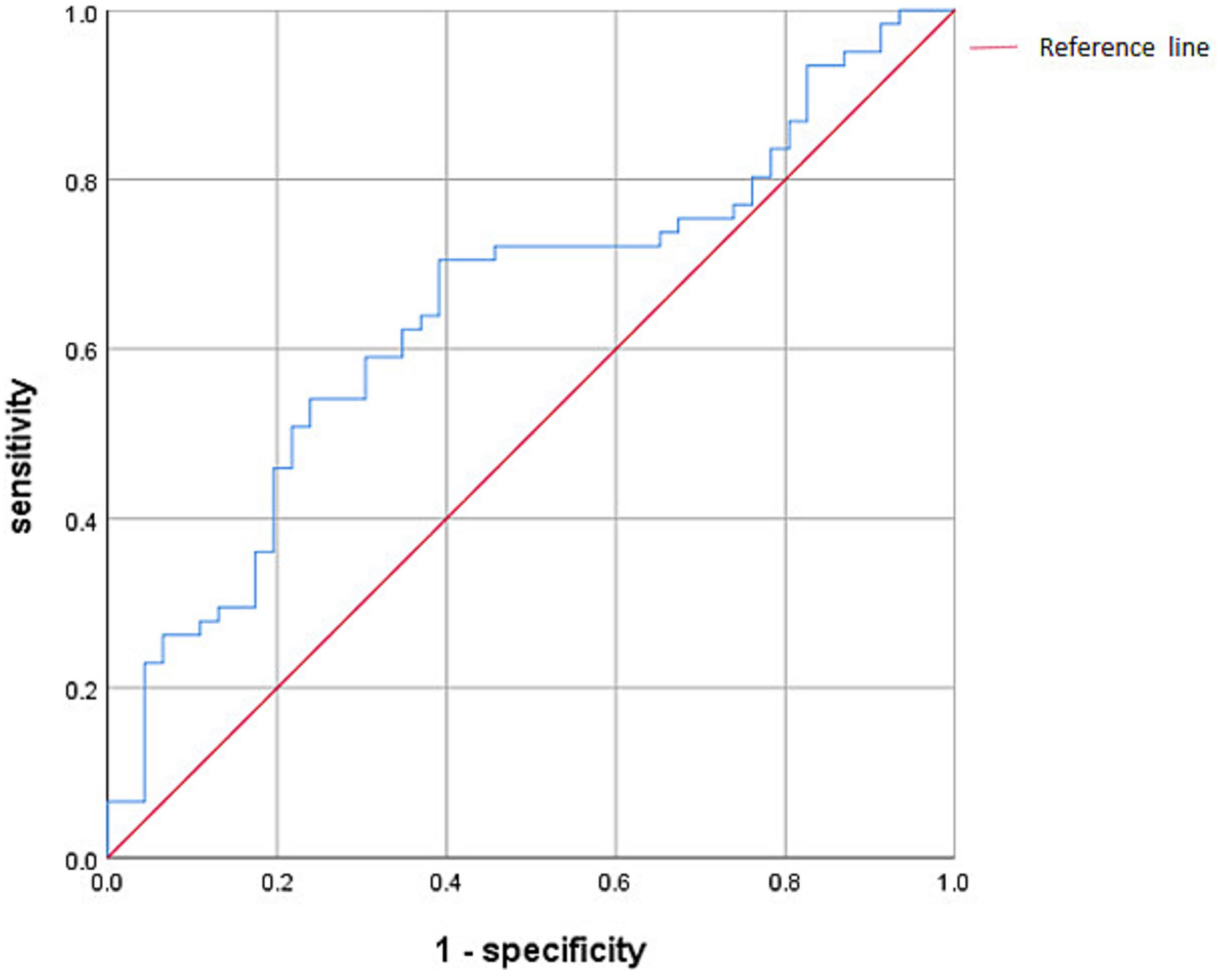
Receiver operating characteristic (ROC) curve analysis of the F/B. the area under the curve was 64.8%, the sensitivity was 70.5%, the specificity was 60.9%.

The ROC curve was drawn using the F/B, and the ROC-AUC was 64.8%, the sensitivity was 70.5%, the specificity was 60.9%, and the F/B was 4.3 (Figure 7).

**Figure 8:**
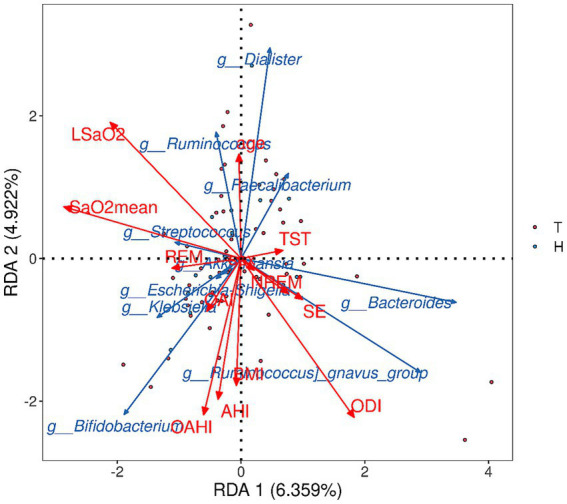
RDA analysis of the top 10 gut microbiota with average abundance at the genus level and sleep monitoring results in primary snoring group and OSAHS group. Red arrows represent PSG indicators and blue arrows represent gut microbiota; when the angle is acute, the two factors are positively correlated; when the angle is obtuse, the correlation is negative; the longer the ray is, the greater the effect of the factor.

Based on the results of species analysis, the top 10 gut microbiota with average abundance at the genus level in the primary snoring group and OSAHS group were selected for RDA. The results showed that RDA1 was 6.359% and RDA2 was 4.922%. The Envfi test showed that only ODI was positively correlated with the abundance of gut microbiome (RDA1 = 0.68, *p* = 0.014). LSaO_2_, SaO_2mean_, OAHI, AHI and BMI were negatively correlated with the abundance of gut microbiome (RDA1 = −0.79, *p* < 0.01; RDA1 = −0.98, *p* < 0.01; RDA1 = −0.31, *p* = 0.011; RDA1 = −0.23, *p* = 0.032; RDA1 = −0.07, *p* = 0.045), and the other indicators were not statistically significant with the abundance of gut microbiome (Figure 8).

**Figure 9:**
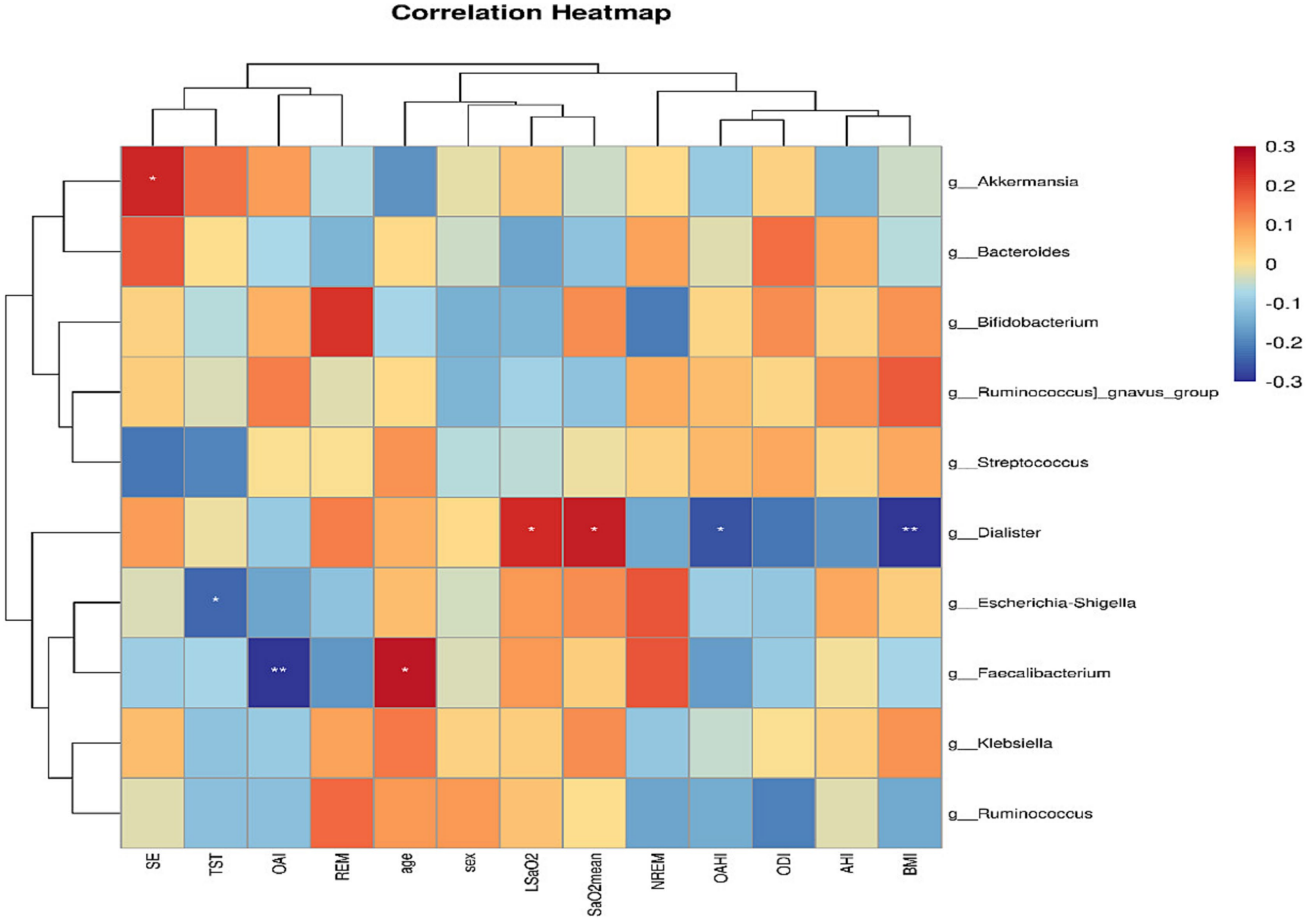
Spearman correlations of the top 10 gut microbiota with average abundance at the genus level and sleep monitoring results in primary snoring group and OSAHS group. The *r* values are represented by gradient colors *r* values are expressed in gradient colors, with darker colors showing higher correlations. Red cells indicate positive correlations and blue cells indicate negative correlations. *p < 0.05, and **p < 0.01.

Spearman analysis showed that at the genus level, Akkermansia was positively correlated with SE (*r* = 0.24, *p* = 0.03). Dialister was positively correlated with SaO_2mean_ and LSaO_2_ (*r* = 0.25, *p* = 0.03; *r* = 0.24, *p* = 0.04) and negatively correlated with BMI and OAHI (*r* = −0.31, *p* = 0.01; *r* = −0.26, *p* = 0.02). Escherichia-Shigella was negatively correlated with TST (*r* = −0.24, *p* = 0.04). The genus Faecalibacterium was positively correlated with age (*r* = 0.27, *p* = 0.02) and negatively correlated with OAI (*r* = −0.33, *p* < 0.01). There was no significant correlation between the remaining gut microbiota and sleep monitoring data (Figure 9).

## 4 Discussion

In this study, we found that the abundance of gut microbiome in children with OSAHS was changed compared with that in healthy children. Focusing on the correlation between gut microbiome and PSG indicators, it was found that some types of gut microbiome changed with the severity of OSAHS.

There was no significant difference in alpha diversity and beta diversity among the three groups, indicating that the number, diversity and evenness of the gut microbiome were basically the same. This is similar to the results of previous studies in adults, and is in contrast to the results in animal models (9–12). It was assumed that the animal model was only induced by chronic intermittent hypoxia (CIH) or sleep fragmentation (SF) mediated changes in gut microbiome under the control of the environment, dietary habits and other factors. However, OSAHS in human adults and children leads to pathophysiological changes such as CIH and SF, and there is no single factor mediating the changes in gut microbiome.

Further comparison between the mild OSAHS group and the moderate to severe OSAHS group showed that the observed otus index and Chao 1 index were statistically significant, indicating that the number of species in the moderate to severe OSAHS group was lower than that in the mild OSAHS group. Under the application of Jaccard and unweighted UniFrac distance methods, the distance difference between samples was statistically significant, indicating that there was a significant difference in species composition between the two groups when only considering the presence/abundance of taxa. The results suggest that the number and abundance of gut microbiome in children with OSAHS decrease with the severity of disease.

The composition of gut microbiota varies among individuals and is relatively unique at the genus and phylum levels (13). Therefore, this study focuses on the analysis of the composition at the phylum and genus levels. At the phylum level, the OSAHS group was similar to a study by Valentini et al. (14) but the group differed at the genus level. This may be due to differences in grouping, study design, geographical location, and other factors.

It was found that the F/B gradually increased with the appearance of OSAHS in our study, which is different from Moreno-India’s study (12). An increase in the F/B is a marker of structural changes in the gut microbiota in obese individuals (15). Several studies have found that these changes may promote weight gain and increase energy absorption (16, 17). Kahleova et al. (18) found that the smaller the reduction in *Bacteroides fragilis* in the Bacteroidetes phylum, the greater the reduction in body weight, fat mass and visceral fat. Therefore, it remains to be seen whether oral probiotics can be used in the future to adjust the F/B and thereby reduce the potential risk of obesity in children with OSAHS.

We found that as ODI increased, the overall abundance of gut microbiota gradually increased according to RDA. This may be related to the fluctuation of oxygen saturation caused by CIH. As fluctuation increases, some gut microbiome increase to adapt to changes in the internal environment. However, as OSAHS worsens, the overall intestinal dominant flora gradually approaches anaerobes and facultative anaerobes, eventually leading to a decrease in gut microbiome abundance.

Using Spearman analysis, we found that some gut microbiota were correlated with sleep monitoring indicators at the genus level, but the degree of correlation was not high. These gut microbiome were associated with metabolic diseases related to OSAHS (15, 19–22). Therefore, it is suggested that the changes in the gut microbiome in OSAHS play a mediating role in related metabolic diseases.

The gut microbiota produces short-chain fatty acids (SCFAs), including butyrate, propionate, and acetate, through the fermentation of dietary fiber. Both OSA and SCFAs are associated with the development of hypertension (23, 24). Hypertensive patients demonstrate an increased F/B, concomitant with reductions in butyrate-producing bacteria (25). Butyrate can maintain intestinal barrier function by upregulating the expression of mucin-associated genes (MUC1-4) in intestinal epithelial goblet cells (21, 22). Kahleova et al. (18) found that the genus Faecalibacterium was positively correlated with butyrate levels. Tang et al. (26) found that the levels of Faecalibacterium were significantly decreased in patients with type 2 diabetes mellitus complicated by OSAHS. In our study, as the severity of OSAHS increased, the abundance of Faecalibacterium gradually decreased. Unfortunately, the content of SCFA was not detected in this study, so we speculate that OSAHS changes gut microbiome and indirectly affects the metabolism of gut microbiome.

In this study, it was found that the ROC-AUC of Firmicutes and the F/B was more than 50%. Although both of them have certain predictive value, the accuracy and specificity are not high, indicating that the detection of Firmicutes and the F/B still have limitations in predicting OSAHS. It is speculated that on the basis of PSG diagnosis, gut microbiome detection has a certain auxiliary diagnostic value.

The study has some limitations. First, this study has a limited sample size and is a single-center investigation, thus warranting cautious interpretation of findings. Second, we excluded the influences of antibiotic, probiotic usage, obesity, and gender, but did not account for the effects of dietary habits and circadian rhythm on gut microbiota. These factors will be incorporated into statistical models as potential confounders in future analyses. Third, without quantitative and qualitative analysis of related serological indicators and metabolomics, the changes in related metabolomics caused by OSAHS by affecting the gut microbiome cannot be shown in detail. Finally, we adopted a cross-sectional study design and did not compare the gut microbiome of children with OSAHS before and after treatment. In the future, we will collect and follow up more data of children with OSAHS to further explore the connection between OSAHS and the gut microbiome.

In conclusion, OSAHS caused varying degrees of changes in the gut microbiome in children. At the genus level, some gut microbiome were correlated with SE, SaO_2mean_, LSaO_2_, TST, and OAI. The ROC curve showed that Firmicutes abundance and the F/B had limited predictive capability for the diagnosis of OSAHS on the basis of PSG diagnosis. Further research is needed to elucidate the relevant mechanisms.

## Data Availability

The raw data supporting the conclusions of this article will be made available by the authors, without undue reservation.
