# Supplementary material for: Preliminary analysis of gut microbiome characteristics in children with obstructive sleep apnea hypopnea syndrome
Source: Front Neurol. 2025 Nov 14;16:1615891. doi: 10.3389/fneur.2025.1615891 (PMC12660262; doi:10.3389/fneur.2025.1615891)
Supplement: Supplementary file 1 [file Table_1.DOCX]

Supplementary Material

Preliminary analysis of intestinal flora characteristics in children with obstructive sleep apnea hypopnea syndrome

Zhihui Wang^1,2†^, Luting Zhou^1#^, Yanyu He^1^, Xueyun Xv^1^, Meng Lv^1^, Zhen Zhang^1^, Fengqian Wang^1^, Shuqi Wang^1^, Yuqing Wang^1*^

^1^ Department of Respiration, Children’s Hospital of Soochow University, No.303 Jing De Road, Suzhou 215003, China

^2^ Jinan Maternity And Child Care Hospital Affiliated to Shandong First Medical University, No.22029, Jing Shi Road, Jinan 250000, China

*** Correspondence:**Corresponding Author: Yuqing Wang
email: wang_yu_qing@126.com

^†^The author is first authorship.

^#^ The author is co-first authorship.

## Supplementary Figures

**
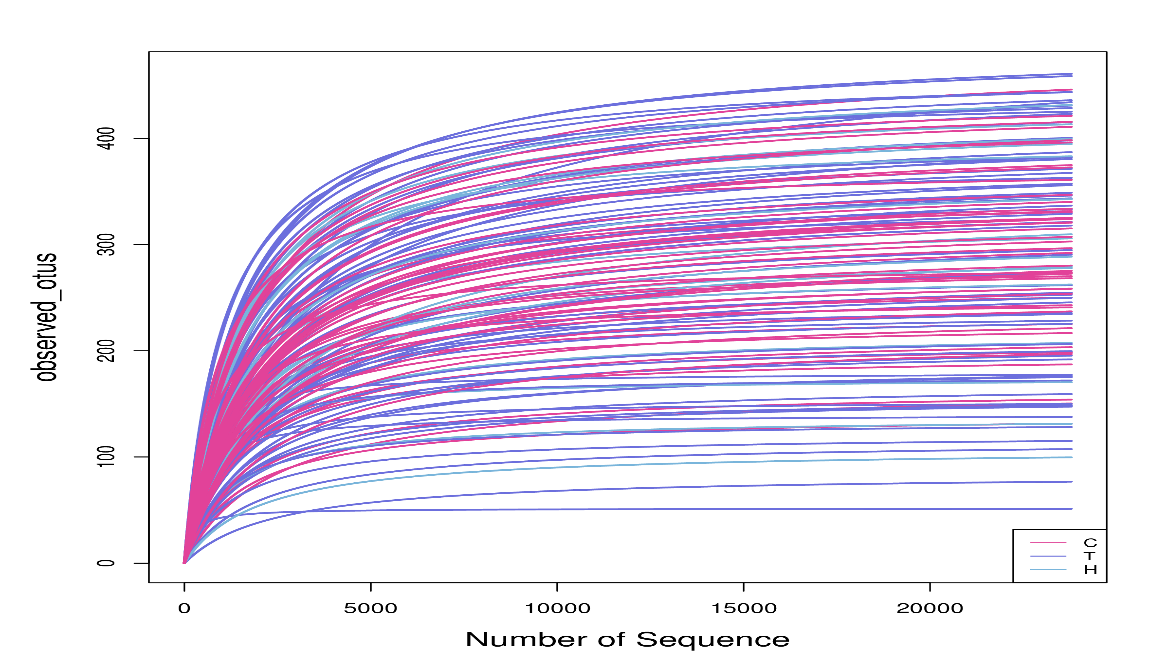
**

**Supplementary Figure 1.** Rarefaction Curve: The curve directly reflects the rationality of the detected sequencing data and indirectly reflects the richness of species. The curve of each sample tended to be flat, indicating that the amount of measured data was saturated and the sequencing depth reached a very high level.


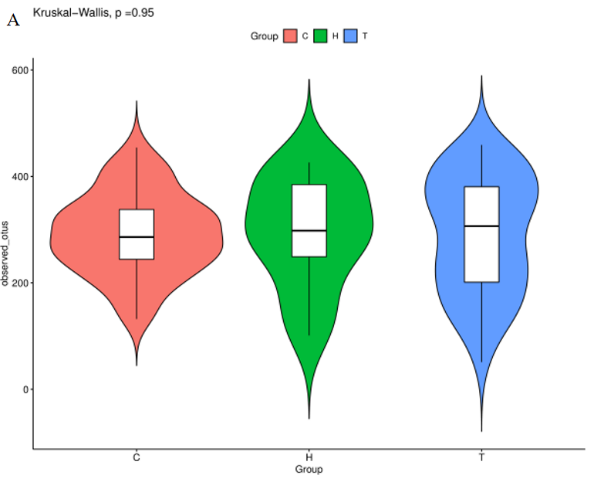

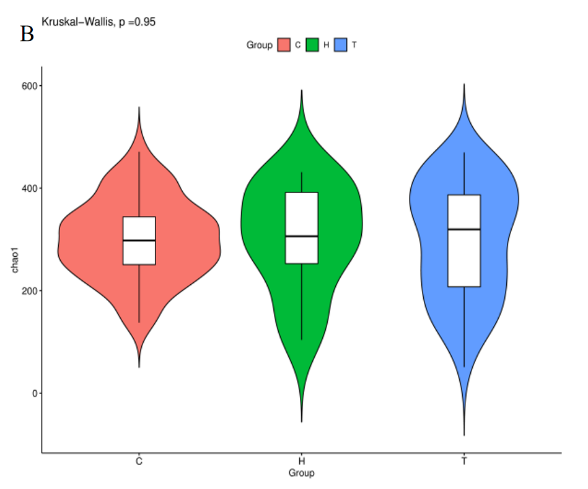


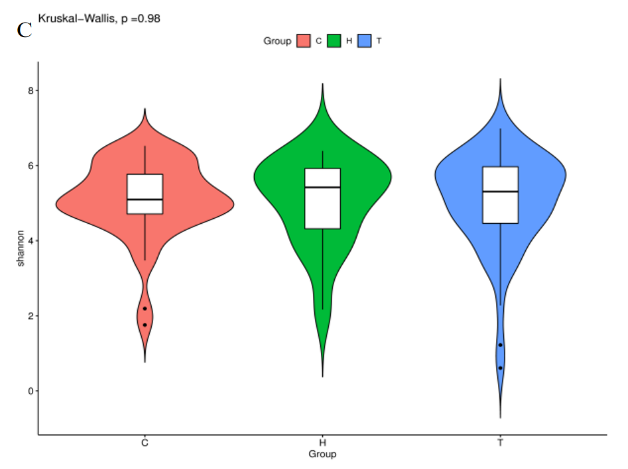

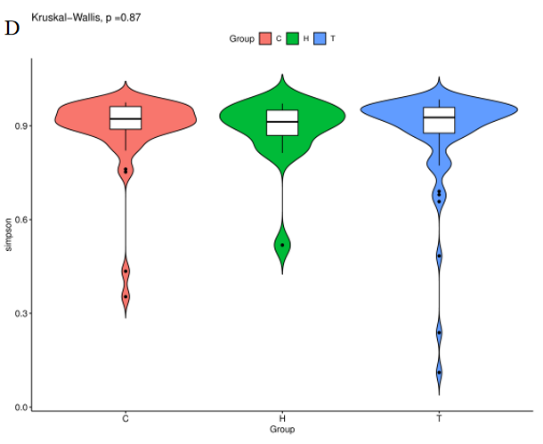


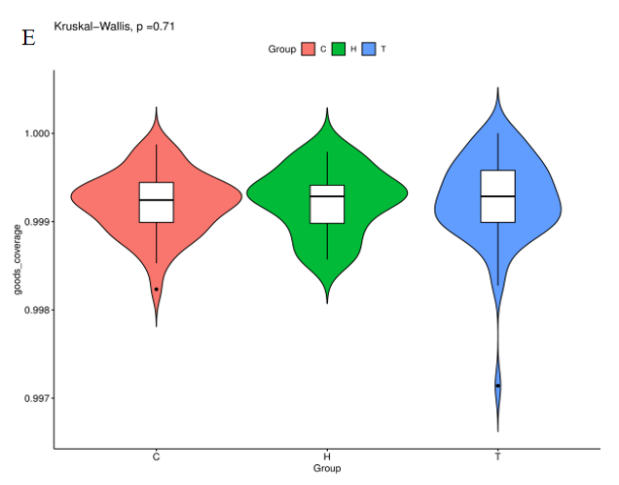

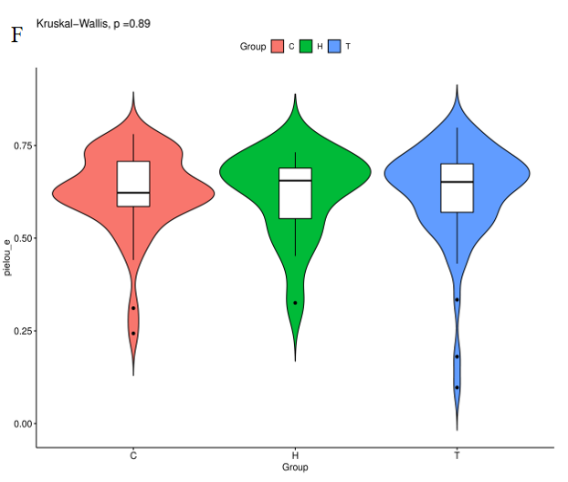


**
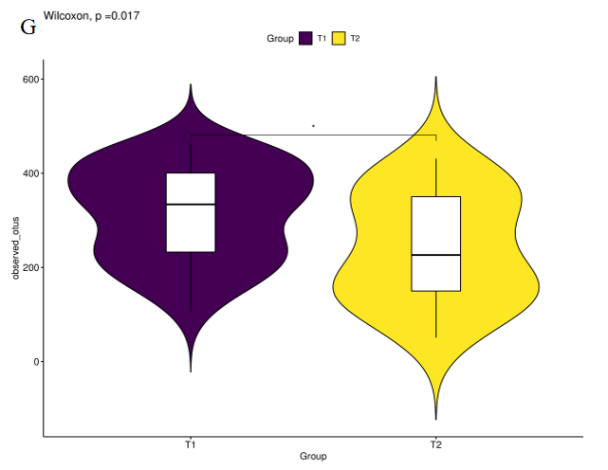

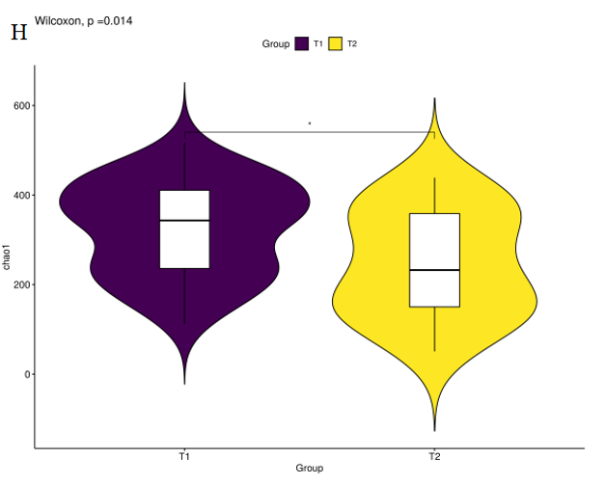
**

**
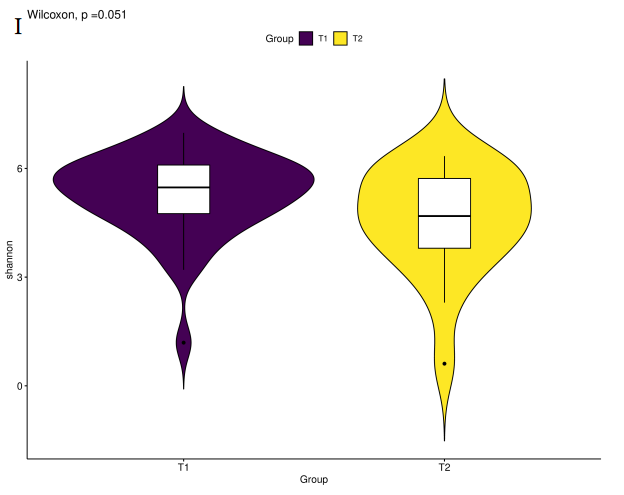

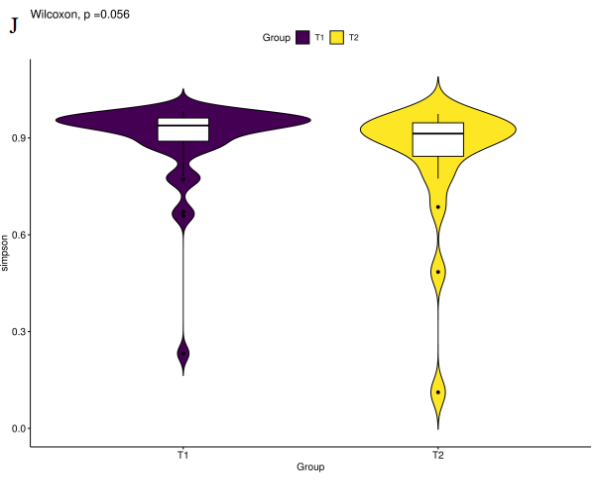

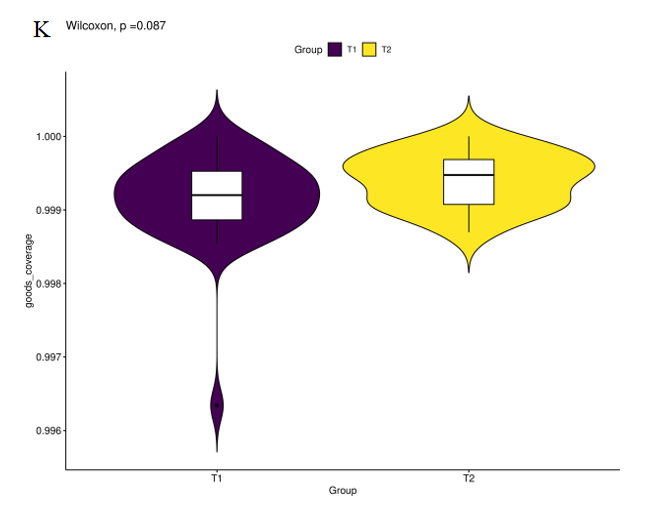

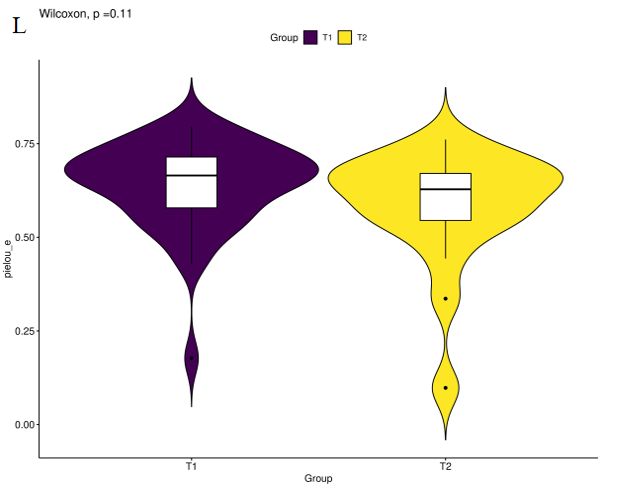
**

**Supplementary Figure 2.**  Analysis of Alpha diversity.

Note: A-F shows the alpha diversity of groups C, T, and H; G-L shows the alpha diversity of groups T1 and T2; C: control group, T: OSAHS group, H: simple snoring group,T1: mild OSAHS group, T2: moderate to severe OSAHS group; ★：P＜0.05.


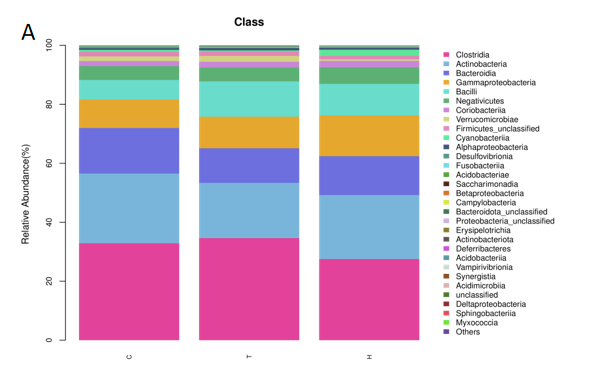


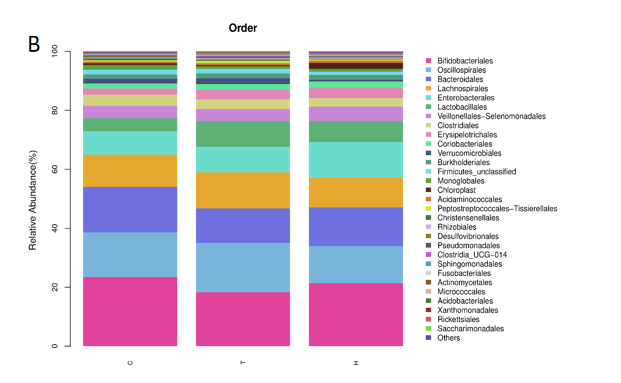


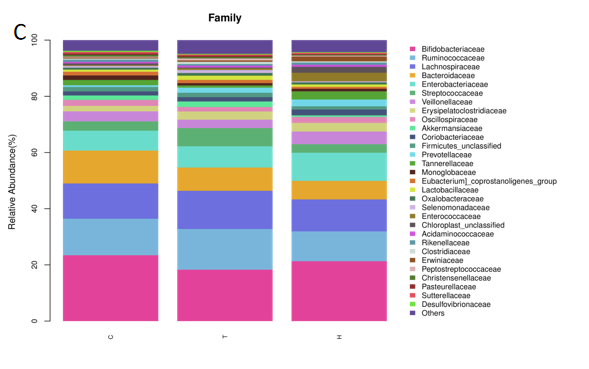


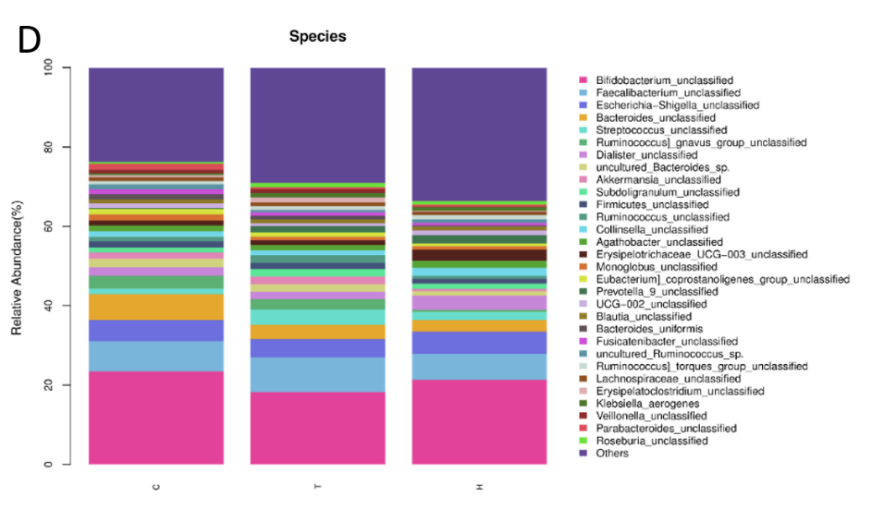


**Supplementary Figure 3.** Histogram of species classification; Class (A), Order (B), Family(C) and Species(D) levels. Note: C: control group, T: OSAHS group, H: simple snoring group; In the figure, the horizontal axis is the grouping, and the vertical axis is the relative abundance of a bacterial group. Different colors correspond to different species at the same level.


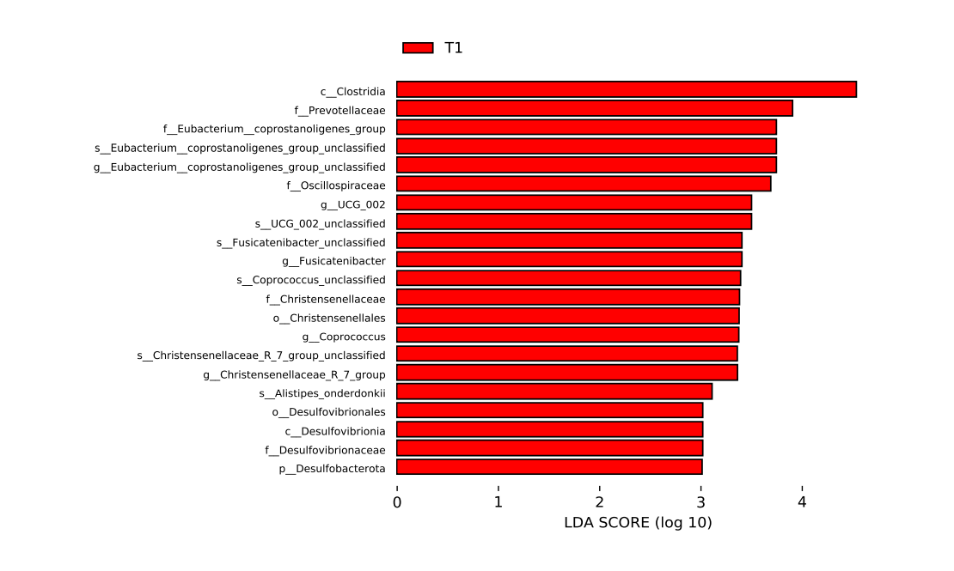


**Supplementary Figure 4.** Histogram of LDA distribution: Comparison of mild OSAHS group and moderate to severe OSAHS group. Note: Relative abundance of the most discriminant fecal metabolites according to LEfSe. T1: mild OSAHS group.
